# Victims of the Terrorist Attacks in Belgium and Professional Mental Health Aid Barriers: A Qualitative Study

**DOI:** 10.3389/fpsyt.2021.638272

**Published:** 2021-07-02

**Authors:** Roel Van Overmeire, Emilie Muysewinkel, Rose-Lima Van Keer, Lara Vesentini, Johan Bilsen

**Affiliations:** ^1^Mental Health and Wellbeing Research Group (MENT), Department of Public Health, Vrije Universiteit Brussel, Brussels, Belgium; ^2^Interuniversity Centre for Health Economics Research (I-CHER), Department of Public Health, Vrije Universiteit Brussel, Brussels, Belgium

**Keywords:** mental health, terrorism, access-barriers, post-traumatic stress disorder, victims

## Abstract

**Introduction:** Terrorist attacks can cause short and long-term stress-reactions, anxiety, and depression among those exposed. Sometimes, professional mental health aid, meaning all types of professional psychotherapy, would be appropriate, but victims often delay or never access mental health aid, even up to a decade after the initial event. Little is known about the barriers terrorist-victims encounter when they try to access professional mental health aid.

**Method:** Using a qualitative design, 27 people exposed to the 22/03/2016 terrorist attack in Belgium were interviewed using half-structured, in-depth interviews, on their experiences with professional mental health aid. A reflexive thematic analysis was employed.

**Results:** Five main barriers for professional mental health aid seeking by victims were found. First, their perception of a lack of expertise of mental health aid professionals. Second, the lack of incentives to overcome their uncertainty to contact a professional. Third, social barriers: people did not feel supported by their social network, feared stigma, or trusted that the support of their social network would be enough to get them through any difficulties. Fourth, a lack of mental health literacy, which seems to be needed to recognize the mental health issues they are facing. Finally, there are financial barriers. The cost of therapy is often too high to begin or continue therapy.

**Conclusions:** This study showed that the barriers for seeking professional mental health aid are diverse and not easily overcome. More mental health promotion is needed, so that there is a societal awareness of possible consequences of being exposed to terrorist attacks, which might result in less stigma, and a quicker realization of possible harmful stress reactions due to a disaster.

## Introduction

A wave of terrorist attacks has struck Europe in recent years: Paris, France on 13/11/2015, Brussels, Belgium on 22/03/2016, Manchester, United Kingdom on 22/05/2017 and even just last year on 19/02/2020 in Hanau, Germany and on 29/10/2020 in Nice, France. Such terrorist attacks can cause short and long-term stress-reactions, anxiety, depression and post-traumatic stress disorder (PTSD) among those directly exposed (1, 2). Furthermore, among the victims of such attacks who can also develop mental health issues, are family members of those directly exposed or killed in such attacks (1, 3). Professional mental health aid (as in professional psychotherapy) can be necessary, but victims of terrorist attacks often delay or never access mental health aid, even up to a decade after the initial event (3–6). This is similar to what has been observed among victims of other forms of trauma (e.g., interpersonal trauma), who also often delay seeking out professional mental health aid (7). Among these victims of other types of trauma, delaying or not accessing mental health care is related to the public knowledge about mental health (i.e., doubts about treatment possibilities), social factors (e.g., stigma) and individual factors such as believing that mental health issues can be solved on their own or financial issues (7, 8). Yet, little is known about the barriers terrorist-victims encounter when they try to access professional mental health aid (9).

Most of what is known about these barriers comes from studies on the 9/11-terrorist attacks in the United States (5, 6, 9–11). These studies found that victims might not access professional mental health aid (i.e., psychotherapy) because of stigma, financial issues, or practical problems (i.e., no time to undergo professional aid).

However, the mental health aid barriers (MHAB) that exist in Europe might be fundamentally different. In contrast to the United States, West-European countries have a universal healthcare system. Because of this, victims of terrorism in Norway, for example, might access mental health care more easily and more often than victims in the United States (3, 12). Bearing in mind the limited literature available on this topic [e.g., (3, 12, 13)], performing sound scientific studies on the MHAB after terrorist attacks in Europe could reveal interesting new perspectives and uncover possible MHAB that might arise after disasters such as terrorist attacks.

In this study we will investigate the MHAB for victims of the 22/03/2016 bombings in the center of Europe, Brussels, Belgium. Belgium has a compulsory health insurance system, which requires that Belgian residents must be affiliated to a sickness fund of their choice. As such, 99% of the population is covered by the health insurance system (14). The mental health care system in Belgium is fragmented, which is part due to the sharing of competences over different policy levels for mental health care (e.g., some parts are managed by federal level, others by the communities). Furthermore, the mental health care system has problems with access and affordable psychotherapy. Finally, while consultations with a psychiatrist are reimbursed, consultations with psychologists have only been reimbursed since March 2019, and this for maximum four visits a year (14).

By investigating the MHAB for victims of terrorism in Belgium, we hope to contribute to an appropriate psychological aid response for victims of terrorism in our country.

## Materials and Methods

### Design

This study focuses on the terrorist attack of 22/03/2016 in Belgium. During the attack, 32 people were killed, and hundreds were wounded through bombings in Brussels airport and a Brussels metro station. Using a qualitative design, participants were interviewed using half-structured, in-depth interviews.

### Participants and Recruitment

This study will look deeper into the accessibility of professional mental health care in witnesses and relatives of deceased victims of the terrorist attack on 22/03/2016, as this group is most likely to develop mental health problems (15–17). With professional mental health aid, we refer in this study specifically to psychiatrists, psychologists, and other professionals involved in providing psychotherapy.

Participants were included if:

(1) They were witness to the terror scene or the aftermath in either the airport or the metro station, or relatives to someone who was killed during the attack, which corresponds to criteria of criterion A of PTSD in the DSM-V to assess a traumatic event (18).
(2) They attributed mental, psychosomatic or behavioral changes that they had or have experienced to the attack (e.g., problems sleeping, constant alertness, flashbacks, stomach problems, black-outs, paranoia, et cetera). People were included if, in their opinion, the change interfered with their functioning in several areas of their life, and this change was long-lasting (longer than 1 month). This was based on criteria F and G of the DSM-V definition of PTSD, which state that the problems last longer than a month, and interfere with social, occupational, or other areas of functioning (18).
(3) They were 18 years or older.

People were excluded if they were physically injured during the attack. It is likely those victims might have already had some sort of mental health aid during the rehabilitation of their physical wounds.

To increase variation in the sample, researchers spread information about the study through several organizations (e.g., victim-organizations, Brussels-airport, and rescue worker organizations). Recruitment and interviews took place between June 2018 and February 2019. Thus, this is before visits to psychologists were reimbursed (14).

Interviews were conducted by the primary researcher, RV. RV is a social health scientist with experience in qualitative research. Participants were informed that they would be interviewed by a researcher concerning their experiences during and after the attack in Belgium of 22/03/2016. RV interviewed 27 participants in-depth, comprising 25 witnesses, and two relatives. The sample population includes eight females and nineteen male respondents. The age ranged between 25 and 60 years old, while the average age was 44.7 years old (see Table 1).

**Table 1:** Characteristics of sample ID, age, gender, profession, prior problems, and time until seeking help.

**ID**	**Age**	**Gender**	**Exposure**	**Prior problems**	**Time until seeking help for attack-related problems**	**Currently on sick leave/different job**	**Currently still going to therapy**
1	39	Female	Witness	None reported	3 months	/	Yes
2	44	Female	Witness	None reported	1 year	/	No
3	44	Female	Witness	None reported	4 months	On leave since the attacks	Yes
4	55	Female	Witness	Burn-out	1–2 weeks (was already in treatment for burn-out)	Working part time since the attacks	Yes
5	57	Male	Relative	None	3 months	On sick leave since attack	Yes
6	50	Female	Relative	None	3 months	Part-time due to attacks	Yes
7	58	Male	Witness	None	3 months	/	No
8	43	Male	Witness	None	1 month	/	No
9	40	Female	Witness	Involved in violent shooting	7 months	On sick leave	Yes
10	43	Female	Witness	Depression	9 months	On sick leave	Yes
11	54	Male	Witness	Involved in violent shooting	1–2 weeks	Had to change job	Yes
12	56	Male	Witness	None	6 months		No
13	44	Male	Witness	None	1 year, 2 months		No
14	59	Male	Witness	Work-accident	4 months	On sick leave	Yes
15	29	Female	Witness	None	1–2 months	/	Yes
16	40	Male	Witness	Burn-out	1 year	Changed job	Yes
17	25	Male	Witness	None	3 months	/	No
18	30	Male	Witness	None	1–2 weeks	/	No
19	47	Male	Witness	None	/	/	/
20	32	Male	Witness	None	/	/	/
21	55	Male	Witness	None	/	/	/
22	48	Male	Witness	None	/	/	/
23	30	Male	Witness	None	/	/	/
24	48	Male	Witness	None	/	/	/
25	28	Male	Witness	None	/	/	/
26	48	Male	Witness	None	/	/	/
27	60	Male	Witness	None	1 year	Has started working less	Yes

*/, not started professional mental health*.

### Data Collection

A semi-structured interview guide was developed by a multi-disciplinary team, mainly consisting of RV, JB, and R-LV, based on a literature study [e.g., (9, 12)] and the researchers' previous experiences with qualitative interviews with vulnerable groups. The interview guide included topics on their exposure to the attacks, and whether or not they sought out help (see Table 2).

**Table 2:** Table of topic list.

1. Description of changes and health in days/weeks/months/years after the attack. 2. When did the victim notice that he/she was experiencing problems? 3. Reaction on these problems? 4. Need for professional aid? 5. Was there professional aid available? 6. How did he/she come into contact with professional aid? 7. How was the experience of accessing professional aid? 8. How would they evaluate the professional aid?

Interviews were conducted at a place of the participant's choosing, which was mostly at their home or a meeting room at their work. They were audio-recorded and lasted, on average, minimum 1 h and maximum 2 and a half hours. Afterwards, no transcripts were returned to research participants for feedback or corrections.

### Analysis

A reflexive thematic analysis was used, whereby themes and relationships between themes were sought out in the responses of the participants (19). The first step was the familiarization with the data. This was followed by coding of the data, performed independently by RV and R-LV. R-LV is an anthropologist and has a PhD in social health sciences, with an expertise in qualitative research. Coding was performed in NVivo 12.0. To allow a higher intercoder reliability, differences in coding were discussed between R-LV and RV until an agreement of coding was reached. Based on this coding, general themes were sought out. These themes were developed based on the codes that were found, thus allowing more reflexivity in what was found, due the themes not being decided in advance (19). The resulting themes were discussed between R-LV, RV, JB, EM, and LV, which included the discussion of the defining and naming of the themes.

### Ethics

All participants were sufficiently informed about the study (e.g., the purpose), their rights (e.g., the guarantee of their privacy), and potential aid channels (e.g., helplines and psychologists), after which they gave their written consent to participate in the study. Information related to the identity of the participants was removed from transcripts as much as possible (e.g., names, address, et cetera). All coded transcripts were saved on a secured server of the Vrije Universiteit Brussel (VUB), to which all authors are affiliated. Furthermore, this study was approved by the Commission Medical Ethics of the UZ Brussels/VUB (B.U.N. 1430201836125).

## Results

Five participants reported mental health problems that had existed prior to the terrorist attack. Two participants reported burnouts, two traumatic stress reaction, and one depression (see Table 1; column “prior problems”).

The results showed that 19 participants pursued therapy on a consistent basis after the terrorist attack, though one of them (ID 18) only went once. For all these participants, the therapy was related to the experience of the attacks. Twelve participants still attended therapy at the time of the interview. Of the latter, five participants are registered on sick leave since the attack. There were only six participants out of 27 able to identify the type of therapy they received (all EMDR-therapy).

All participants faced professional mental health aid barriers, whether or not they eventually got professional mental health aid. Eight participants had not started professional mental health aid at the time of the interviews.

Overall, five main mental health aid barriers were found: lack of expertise of mental health aid professionals, lack of personal incentives, social barriers, mental health literacy barriers, and financial barriers.

### Lack of Expertise of Mental Health Aid Professionals

This barrier concerns how, in this case, participants negatively view mental health professionals' expertise and understanding.

#### Perceived Lack of Expertise of Professionals

Most victims got some form of “first mental health aid” in the week(s) after the attack: ambulatory visits to psychologists at the Center of Mental Well-being (CAW, in Dutch) or visits from crisis-psychologists at work. However, almost all participants denied the professional help offered. Reasons were diverse: a perceived lack of expertise (e.g., the professionals were perceived to be too young to have the proper experience or perceived not having the proper training), in one case, social support felt more helpful.

‘*To me, there is no Victim Aid (the institution that helps people after traumatic events). There are people that mean something – maybe – on paper. Like “Pretend you know something!” (laughs). We got people, but they don't know anything – how would they? They're not trained for this. We had to help them (the people from Victim Aid) when they came'* (ID 5).

This perceived lack was also noticed in the search for proper therapy. Certain participants in the sample ended up with a psychologist or psychiatrist that were unsuited to their needs. Some victims explained that finding a suitable therapist is difficult because there are too few experienced therapists in trauma-care. In addition, there is no proper way to find out who is specialized in trauma-care.

‘*[Therapists] aren't prepared for something like this. And I think there's a huge gap there, both with victim support as with psychologists, that there isn't a specialization in trauma-care. It doesn't exist… […] The processing of the death of B. (deceased) becomes a side issue, you know, they work on other domains, and that's where the problem is, and you feel they don't really know what to do with it' (ID 6)*.

#### Perceived Lack of Understanding of Professionals

Some participants acknowledged to feel a lack of connection with the health provider and preferred talking to fellow victims because of the shared experience. This means that therapists cannot understand the experience of being in a terrorist attack, unless they have experienced it as well, and thus cannot properly help the victims.

‘*I went there (to a psychologist) myself on a certain moment, just to see, look, am I right (that therapy cannot help)? I said a few things, this and that is my problem and I got as answer “Yeah, that's normal, if you still have it in 3 months, come back again”. That's the answer of those professionals. I was kind of disappointed, but yeah, I think – you hear colleagues say, “Those people, they can't help, because they do not understand”. I just went there to test that prejudice'* (ID 18).

### Lack of Personal Incentives

Eight participants never sought out help, regardless of the offered mental health aid. Among these, there were three that wished they had contacted professional mental health aid but did not. Reasons mentioned were the uncertainties, the threshold of contacting a professional solely by themselves, but also the fear of confrontation with what they felt during and after the terrorist attacks.

‘*Sometimes, it's still difficult. […] I don't know with who or what I can talk to about it. […] There were mails, saying that you could contact a number – in the first period, day and night, and you could get into contact with a psychologist. But, like I said, I don't know with who I would come into contact with that number, so I never called'* (ID 19).

Someone who had delayed seeking out professional mental health aid for 4 months, recalled the personal barriers she experienced.

‘*I thought long about that (going to therapy). But to do it… Then you have to take the step to be involved with that. And that's what was hard for me, to be occupied with that. Because – you get confronted again with everything. And, just, you know, if you do it, it's going to be tough, and taking the step on your own to go is already tough. So yeah, you got to do it all again on your own, you know'* (ID 3).

### Social Barriers

Additionally, people are influenced by their environment: family, friends, colleagues. Their reactions or lack of them can also act as a barrier. This translates itself in the way a lack of support by friends and family to seek out help, the trust in the social support or stigma can become mental health aid barriers.

#### Lack of Support by Friends and Family

Interviews showed that almost none of the members of participants' social support network advised them to seek professional help, in part because their social network tried to ignore the events happened. This let participants to believe they could handle their mental health issues, since nobody in their surrounding outed any concern.

‘*After that we just went back to work, and that was it… It was like “It happened, and life goes on”. It was almost like taboo. […] So, when I went to the doctor, and he asked if everything was okay, I didn't even dare to say ‘No, everything is not okay', because you get so little support, and it makes you think “I actually don't want any misery, everything is okay”. And so, he gave me an okay to work'* (ID 10).

‘*I have always kept for myself. Like, I talked about (my problems) with a few people, but then you hear “Yeah, but that's normal, after what you have been through”, while I thought “Huh, that isn't normal… Or maybe it is, and I'm wrong?” It was very confusing'* (ID 2).

In a couple of cases, participants had to go on sick leave, leading to members of their social environment confessing to have received earlier signs of mental distress within the victims.

‘*Then (after going to a mental health aid practitioner) you hear remarks like, “It had to happen, you have to break sometime, you can't be a tough guy all the time, everybody has to go under sometime”*' (ID 12).

#### Stigma

Stigma played a role for a lot of victims in the pursuit of a professional. Some victims confirmed that psychological problems and therapy were indeed connected with stigma. For example, they did not want to admit they had mental health problems because they might appear to be weak or found mental health problems in general to be dubious and/or an excuse.

‘*Everybody has a right on a burn-out these days. There was someone who had to do administrative tasks, and he was suddenly gone: burn-out… And then you get your own mental problems, and you think, they're going to compare me with that guy. You're there with REAL problems, and someone else… So, yeah, psychosocial wellbeing, it has a dirty side to it'* (ID 16).

‘*Psychological problems are still taboo. Nobody wants to get out and show that they go to a psychologist, almost nobody wants to admit they use Sipralexa or another product. You know, since I have openly said that I take it, that there are 4 mothers who admitted to taking Sipralexa. What the fuck? I don't hide it anymore. Sure, the good old Flemish way, but goddamnit, they can see how a person goes through the dirt too. […] Inner scars, you can't see those'* (ID 9).

In addition, one male participant suggested a possible link between stigma and differences in generations as a reason for never seeking professional aid.

‘*I don't think anyone will dare to ask that (for mental health aid). It will be until the next generation of millennials – they tend to be more inclined to being emotional. […] Showing emotions, yeah, it – it's seen as weak'* (ID 26).

#### Trust in Social Support

Interviews revealed that victims not always desired mental help, as they wanted to handle their own problems through talking with friends and family. Some felt it was enough to process the events. This was not related to their perception of a possible lack of understanding or expertise of professionals. These participants felt that they were able to solve any problems they might have on their own. Though this might be connected with stigma, it was not obvious and had perhaps more to do with not wanting to be dependent on other people.

‘*I never went (to the psychologist). I'm not like that. I always try to do it on my own, yeah. […] I think so (that it worked to handle it on his own). By talking about it, with family and friends. I tried to process it like that'* (ID 23).

Some who did seek out professional mental health aid, but were disappointed, also trusted in their social support.

‘*(My psychologist) had his own problems. I did one session, paid 50 euros, to play psychologist myself. His child had died the year before on the day I came, and I started listening to his story. So… I got through it with the help of the people around me. I have a lot of luck with my friends, they're golden, really good friends and they're always there for me'* (ID 15).

### Barrier Due to Mental Health Literacy

Due to a lack of knowledge of mental health consequences of large-scale disasters, some respondents postponed searching for help. This was partly due to an underestimation of the problems they were experiencing, and a lack of knowledge of post-traumatic stress disorder.

#### Knowledge of PTSD

PTSD was for a few participants a whole new concept, including the symptoms related to traumatic events. This seemed to have been a barrier in acknowledging the need for help. While they associated their problems with the attack, they only faced the seriousness of their problems once it was linked to a medical condition whether or not diagnosed by a medical professional (e.g., PTSD).

‘*And then the doctors finally diagnosed that it was post-traumatic stress syndrome. But someone who doesn't have it, can't understand it. I have read a lot about it now, but I had never heard from it before, from that post-traumatic – but you hear the same stories everywhere, the same complaints of people who get little sympathy. Because if you don't have it, you can't understand, impossible'* (ID 11).

#### Delayed Realization

Most participants reported increased aggression, long-lasting sleeping problems, paranoia, constant alertness, black-outs, loss of concentration and in some cases, psychosomatic complaints. However, participants confessed to postpone searching for help regarding their problems, because they often denied or underestimated the scope of their problem(s). In some interviews, a moment of realization of their problems was recounted as: “*This is not me*.” Up until that point victims tended to go about their day as though nothing had happened. This occurred in people without any experience with mental health providers, as well as in participants with previous encounters.

‘*So, we go there (to a mall), and at a certain moment I saw a movement, and then (a child screamed) “Mommy, mommy, mommy”, and those were Americans. And that sounded exactly the same (it remined him of the child he saw sitting next to her/his death mother in the airport after the attack). That was like getting hit in the face, I didn't see anything anymore, I was completely gone, I only saw that mother lying there – from then on they started with EMDR, because they told me “You really need it, man”*' (ID 14).

‘*I got guns at home, and there was screaming on the square in my village, and I thought, what's going on here? So, I took my gun and ammunition, was waiting by the door, and thought, if someone comes in here… But it was nothing. And then I said to myself: “Maybe I should talk with a psychologist*”' (ID 17).

### Financial Barriers

Because of the financial costs of mental health aid, or reimbursement problems for the suffered damages after the terrorist attack, participants experience difficulties accessing or continue accessing mental health care.

#### Financial Costs

During the interviews, some victims mentioned the cost of psychologic therapy as a reason for limited or no accessibility to mental health aid. In almost every case, this regarded to psychologists, as psychiatrists are reimbursed by the health insurance funds in Belgium. Patients' preference to seek help from a psychologist (viewed as a mental health professional that listens to patients), rather than a psychiatrist (perceived to be only prescribing medicine), forms an additional insight, and makes financial costs a relevant barrier.

‘*So, I just went to get 5 sessions of EMDR. And that helped - kind of. But not enough to like function normally. Ehm… Then there was a problem with those sessions, you know, it was a trauma psychologist, they're not reimbursed, and so that was 100 euros per session. So, in a week, I went 5 times, or in two weeks, and that was 500 euros. That's a lot, at least for me. I couldn't keep doing that, so I stopped it. I thought it'd be okay, but it wasn't'* (ID 11).

#### Insurances

Half of the people in the sample encountered insurance problems: a lack of reimbursement due to inadequate invalidity-ratings from insurance doctors, difficulties in reading the insurance papers (e.g., in French, or unreadable because of legal jargon), receiving a different diagnosis from the insurance doctor or not getting recognized as a victim of a terrorist attack. The latter leads to increased pressure on the individual's finances and leads to problems in accessibility of mental health care.

‘*It is confronting and difficult that you get into a situation where your child died and was actually killed, but you still have to prove that (he/she) is a victim. And that you yourself are a victim. And then you find the hardness of insurance companies, who of course think about their own wallet'* (ID 6).

The interviews of some participants reflect a reluctance to filling out insurance paperwork. Some attributed this feeling to a sense of reliving the situation when completing those forms. While others found the insurance paperwork solely too complex in order for them to fill them out.

‘*You have to make an estimation of the costs. Yeah, but how can you know beforehand? All those medical costs? And I think that the insurances handled that way too quickly. I mean, for us – it might not be financial problem, because we have money, but others without a doubt will have problems. Yeah… People just have to find their way on their own, which basically means the insurances want to ignore them (because the process is so complex and difficult). I just filled in the papers – they have been here for a half a year, because it was just so intimidating, like “How do I start this?”*' (ID 2).

## Discussion

As far as we know, this study is the first study attempting to provide more insight in professional mental health aid barriers for victims of terrorist attacks, using a qualitative design. Over the course of 27 interviews, a wide variety of reasons were found as to why victims of terrorist attacks may oppose seeking mental health care or continuing seeking mental health care. It became quite clear there is an overall lack of awareness of mental health problems in this context. Victims feel held back by stigma, their social environment, a lack of personal incentives, financial problems, their lack of mental health knowledge, or just prefer to handle their problems on their own. It also appears multiple barriers can arise within one individual.

As in other studies, social support played an important role in accessing professional help (5). While some findings in this study point to the positive role social support can play in accessing mental health aid, there is also clear evidence of victims holding back of reaching out for professional help, due to their social environment. A possible explanation for this phenomenon can be attributed to the conflict between one's own awareness of mental health concerns and the necessary acknowledgment of the mental health concerns by family and friends to initiate the search for professional care. Such lack of acknowledgment of important loved ones may also explain why other studies found that many victims opt to handle their own problems (9).

In addition, psychological trauma-related symptoms are not directly connected to seeking help of a mental health professional (20). Even although participants confessed to be aware of one's problems, they admitted it took some time before they really acknowledged the severity of their condition. A possible reason might be their lack of mental health literacy to correctly assess mental problems. As a consequence, they tried to uphold the social role they employed before the attack (e.g., tried to continue work, be mother, etc.). Only the severe impairment of their social roles by their mental health issues (e.g., break-down due to flashback, or almost shooting someone because of the increased perception of threat), gives them the incentive to go to psychotherapy.

The role of the universal healthcare system is complex. As Stene and Dyb (12) pointed out, access to specialized aid in countries such as Belgium is less income-driven compared to countries such as the United States. It is true that psychiatrists are reimbursed in Belgium. However, this was not the case for psychologists at the time of data-collection, which ended in February 2019. Since March 2019 psychologists are reimbursed, but only for a maximum of four times a year (14). This is problematic as psychiatrists are often perceived as the ones people consult specially to get their prescribed medication, while psychologists are the actual specialists with whom they can share their burden (and hope to solve their problems via psychotherapy). Thus, while the universal healthcare system portraits an accessible institution in terms of costs, people often are limited in finding affordable mental health service in terms of an emotional connection. Additionally, insurance companies in Belgium play a dubious role in supporting victims of terrorist attack in seeking help. Besides not giving the full financial compensation to stimulate victims' mental health aid seeking behavior, insurance companies are even entitled to refuse any recognition to victims' problems, which then creates an extra mental barrier of recovery (21). Victims then feel as if the experience is downplayed as well as their correlated problems, ultimately leading to stigma, a previously mentioned barrier in seeking mental health aid, resulting in even more problems for the individual.

Furthermore, a structural barrier exists due the lack of knowledge in Belgium on the long-term relationship between mental health issues and disasters. First, different from some other countries such as the U.S., where PTSD is culturally a widespread concept (22), the initiatives regarding trauma care in our country are relatively new (23). Second, there is no record of a certified and publicly accessible network of psychotrauma therapists, as stated in the report on the attacks of 26/03/2020 by the Belgian Federal Government (24), which makes it practically significantly more difficult to find suitable mental health care. Also, other authors point to the importance of knowing one's way through the sometimes complex mental health aid network in finding smoothly the right care when needed (9, 10).

Given this insight, regardless of the information spread proposing therapy treatments for people dealing with the consequences of a traumatic experiences (e.g., cognitive-behavior therapy or EMDR-therapy), this knowledge seldom finds its way to public health studies or policy (25). The consequences of a gap in knowledge might manifest itself in consulting unexperienced or underqualified psychologists or psychiatrists, who might not be able to properly diagnose a disorder. Yet, gaining recognition for possible post-traumatic symptoms is important for victims (26).

The first recommendation based on this study is to invest as society in more profound and adequate mental health promotion. This should include the fact that the majority of people exposed to traumatic events do not develop mental health issues of any kind. If immediate emotional reactions do occur, they mostly fade over a timespan of 1 month (e.g., acute stress disorder). The latter entails there is no point in publicly advocating the necessity for everyone exposed to terroristic attacks to seek out mental health aid (22). People, in general, are far more resilient than most studies acknowledge (27). Thus, such campaigns should be aimed at awareness of possible long-term problems, but that short-term reactions are normal. Furthermore, while there is often a focus on long-term disorders such as PTSD, other diagnoses are still possible after such disasters, such as depression or anxiety disorders (5).

A second recommendation can be made regarding the need of more available centers for victims of traumatic events. France has already integrated such a center for victims of trauma (28), while in the United States there is Project Liberty, a large intervention program established after 9/11 (29). This project has shown to increase service access in vulnerable groups (30). Centers such as the Project Liberty not only support victims, but also contribute to informing them on mental health consequences (31). This is not to say that Belgium goes without any centers for victims of traumatic events, such as e.g., the “Health center after sexual violence” (in Dutch: “Zorgcentrum na Seksueel Geweld” linked to the University Hospital of Ghent). However, there remains in gap for general traumatic events. In term of costs impact, it seems also in the best interest of our society to invest in such centers compared to the costs of employees on sick-leave due to mental health issues, or the costs of long-term psychiatric care. Such centers have the great advantage that those exposed do not have to see different therapists to find a suitable therapy, as these centers have the necessary information, and the most suitable, evidence-based therapy. Furthermore, these centers being publicly known, improves to combat the stigma that might be associated with the mental health problems. The need for such centers for an event such as terrorist attacks, is confirmed by the victim-support groups that have arisen after the 22/03/2016 attacks in Belgium (e.g., V-Europe, Life4Brussels…).

This study entails several limitations. A recall bias is possible as the interviews took place 2.5–3 years after the attacks in Belgium (22/03/2016). Furthermore, the uneven distribution among our participants of gender, with a majority of men, could possibly have affected the reported results. Third, only two relatives of people who died during the attack could be included in our research population, despite the fact that relatives can provide more insights. Fourth, education level plays a role in knowledge about the mental health care landscape, but was not included in this study. Nevertheless, this study does embark on in-depth information regarding an underexposed topic, namely mental health aid barriers after terrorist attacks.

To conclude, the insights of this study could provide a positive incentive to form an adequate psychological support framework for victims of terrorist attacks in Belgium and other European countries. The goal of further research should not only be to investigate the possible necessary political interventions in context of mental health and terroristic attacks, but also increase the focus on constructing more resilience.

## Data Availability Statement

The raw data supporting the conclusions of this article will be made available by the authors, without undue reservation.

## Ethics Statement

The studies involving human participants were reviewed and approved by UZ Brussels/VUB Ethics Committee. The patients/participants provided their written informed consent to participate in this study. Written informed consent was obtained from the individual(s) for the publication of any potentially identifiable images or data included in this article.

## Author Contributions

RV and R-LV analyzed the data. RV and EM wrote first draft together. All authors contributed to the discussion of the themes. All authors read and corrected the drafts.

## Conflict of Interest

The authors declare that the research was conducted in the absence of any commercial or financial relationships that could be construed as a potential conflict of interest.
